# Bench-to-bedside strategies for osteoporotic fracture: From osteoimmunology to mechanosensation

**DOI:** 10.1038/s41413-019-0066-7

**Published:** 2019-08-15

**Authors:** Yong Xie, Licheng Zhang, Qi Xiong, Yanpan Gao, Wei Ge, Peifu Tang

**Affiliations:** 1 Department of Orthopedics, Chinese PLA General Hospital, Beijing, China; 2 Department of Oncology, Chinese PLA General Hospital, Beijing, China; 3 State Key Laboratory of Medical Molecular Biology and Department of Immunology, Institute of Basic Medical Sciences, Chinese Academy of Medical Sciences, Beijing, China

**Keywords:** Bone, Bone quality and biomechanics, Metabolism

## Abstract

Osteoporosis is characterized by a decrease in bone mass and strength, rendering people prone to osteoporotic fractures caused by low-energy forces. The primary treatment strategy for osteoporotic fractures is surgery; however, the compromised and comminuted bones in osteoporotic fracture sites are not conducive to optimum reduction and rigid fixation. In addition, these patients always exhibit accompanying aging-related disorders, including high inflammatory status, decreased mechanical loading and abnormal skeletal metabolism, which are disadvantages for fracture healing around sites that have undergone orthopedic procedures. Since the incidence of osteoporosis is expected to increase worldwide, orthopedic surgeons should pay more attention to comprehensive strategies for improving the poor prognosis of osteoporotic fractures. Herein, we highlight the molecular basis of osteoimmunology and bone mechanosensation in different healing phases of elderly osteoporotic fractures, guiding perioperative management to alleviate the unfavorable effects of insufficient mechanical loading, high inflammatory levels and pathogen infection. The well-informed pharmacologic and surgical intervention, including treatment with anti-inflammatory drugs and sufficient application of antibiotics, as well as bench-to-bedside strategies for bone augmentation and hardware selection, should be made according to a comprehensive understanding of bone biomechanical properties in addition to the remodeling status of osteoporotic bones, which is necessary for creating proper biological and mechanical environments for bone union and remodeling. Multidisciplinary collaboration will facilitate the improvement of overall osteoporotic care and reduction of secondary fracture incidence.

## Introduction

The major characteristic of osteoporosis is a decrease in bone mass and quality,^1^ rendering people prone to osteoporotic fracture (fragility fracture) caused by low-energy trauma.^2^ Osteoporosis is a prevailing skeletal disease of the elderly; nearly 200 million osteoporotic patients are diagnosed annually, and almost 9 million osteoporotic fractures occur worldwide.^3^ Surgery is the primary treatment strategy for osteoporotic fracture; however, poor prognoses are presented due to the combination of biological and surgical factors.^4^ The common sites of osteoporotic bones are usually compromised and comminuted, which makes it hard to achieve an optimum reduction and stable fixation.^3,5^ Osteoporotic fractures occur mostly in elderly patients, who exhibit underlying, unfavorable systemic conditions that are prone to complications.^6^ The abnormal remodeling status of bone with osteoporosis would deteriorate after bed braking, which poses a disadvantage with respect to fracture healing and bone callus strength; furthermore, the refracture risk following surgery increases significantly.^7^ In terms of the complexity of treatment and poor prognosis, the annual facility-related hospital cost of osteoporotic fractures is the highest (up to $5.1 billion), followed by that of myocardial infarction and stroke.^8^

Although the results of the clinical studies remain controversial, the majority have demonstrated that decreased callus area (20%–40%) and bone mineral density (BMD) occur in the fracture sites of elderly osteoporotic patients^4^. Studies have indicated that the delayed or nonunion of osteoporotic fractures is implicated in the scarce capacity of bone regeneration with aging.^9,10^ Additionally, the bone properties of such patients are quite different from those of normal individuals and are manifested in the decrease of bone mechanics and mechanosensation, as well as the abnormal bone metabolism caused by immune disorders.^11^ To improve the current unsatisfactory status of osteoporotic fracture treatment, we must first gain an in-depth understanding of the mechanism of fracture healing in elderly patients with osteoporosis. Herein, we highlight the pivotal roles of mechanical loading and osteoimmunology in aging-related osteoporotic fractures, guiding the intervention in osteoporotic fracture patients combined with an optimal treatment strategy for improving the overall standard of care and reducing the incidence of secondary fracture.

## Static and dynamic changes in osteoporotic bone

**Fig. 1:**
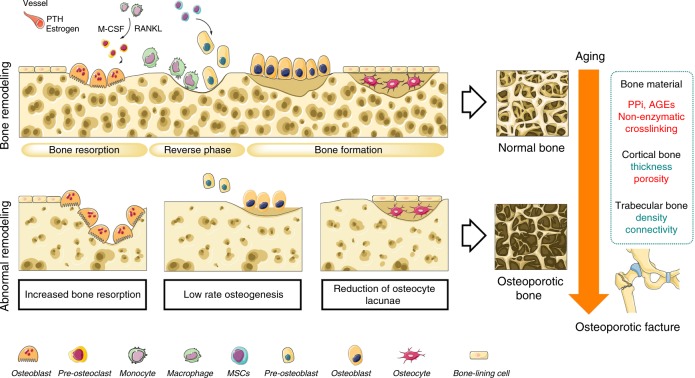
Static and dynamic changes in osteoporotic bone. An osteoporotic fracture is the macroscopic result of microstructural alterations that change the response of bone to the applied load. The aging process in osteoporotic bone would lead to overaccumulation of PPi, AGEs, and nonenzymatic crosslinking of collagen, which disturb the normal organization of bone material. With the increase of bone resorption and low rate osteogenesis, the osteocyte lacunae reduction leads to decreased trabecular thickness and more porous cortical bone. PTH parathyroid hormone, M-CSF macrophage colony-stimulating factor, RANKL receptor activator of nuclear factor kappa-B ligand, PPi inorganic pyrophosphate, AGEs advanced glycation end-products, MSCs mesenchymal stem cells. “Red” refers to upregulation; “Green” refers to downregulation

Bone is a unique tissue due to its elasticity and strength that permits deformation under a certain level of loading stress before failing.^12^ The strength of bone is mainly dependent on the distribution and density of the inorganic matrix mineralization.^13^ Cortical bone consisting of dense and well-organized lamellae has higher strength but a lower capacity to withstand a load that exceeds the elastic deformation range compared with that of trabecular bone, which is composed of unparallel lamellar units with variable porosity (50%–90%).^14^ The mechanical competence of cancellous bone is based largely on the BMD, while the stiffness of cortical bone is highly dependent on its porosity.^3,15^ In contrast to calcified matrix mineralization, the organic matrix (e.g., collagen and noncollagenous proteins) is thought to control bone ductility and its capacity to withstand an impact without cracking.^16^ A large proportion (90%) of the organic matrix is composed of type I collagen, which undergoes numerous posttranslational modifications.^17^Among them, enzymatic modifications positively affect the biomechanical stability of bone, while nonenzymatic crosslinking is associated with a deterioration in these properties.^16^ Noncollagenous proteins, including osteopontin (OPN) and osteocalcin (OCN), account for 10% of the organic matrix and limit crack energy through the control of hydroxyapatite size and orientation.^18^ Whereas bone material properties provide only a static snapshot of bone quality, the abilities of self-regeneration and remodeling provide a dynamic profile of bone health.^19^ The cortical and trabecular bone both undergo lifelong remodeling coupled with bone resorption, which is mediated by osteoclasts following osteoblastic bone formation.^20^ Osteoclasts are of hematopoietic stem cell (HSC) origin and share precursors with macrophages.^21^ In the presence of macrophage colony-stimulating factor (M-CSF), osteoclast precursors differentiate to preosteoclasts by the binding of receptor activator of nuclear factor kappa-B ligand (RANKL) to its cognate receptor, receptor activator of nuclear factor kappa-B (RANK). These mononuclear preosteoclasts then fuse to form multinuclear bone-resorbing osteoclasts.^21^ In contrast, osteoblasts are derived from mesenchymal stem cells (MSCs),^22^ and osteoblastic bone formation is separated from resorption by a reversal phase for several weeks.^23^ Mature osteoblasts then differentiate into osteocytes, which reside in small lacunae inside the calcified bone matrix.^24^ The long dendritic extensions of osteocytes together with the cell bodies form the lacuno-canalicular network (LCN), which allows direct signal transduction. The speed of mineral accumulation in the bone remodeling cycle is also affected by numerous endocrine factors, such as parathyroid hormone (PTH) and estrogen, which are supplied by the bone vascular systems.^25,26^ However, the normal regulation of bone remodeling could be interrupted as a consequence of skeletal senescence,^27,28^ which impact the integrity and biomechanical properties of both cortical and cancellous bones.^29^ The abnormal bone remodeling shifts toward bone resorption, which is either due to excessive activation of osteoclasts or to a low capacity of bone regeneration.^30^ In addition, age-related loss of proteostasis and increased levels of oxidants result in the overaccumulation of inorganic pyrophosphate (PPi) or advanced glycation end-products (AGEs). During the development of osteoporosis, osteocyte numbers per unit of bone area are gradually reduced,^31^ resulting in decreased trabecular thickness and more intracortical porosity.^32^ These considerable changes in the matrix composition and structure cause deterioration of bone quality and compromise its resistance to mechanical loading.^33^ Thus, osteoporotic fractures are the macroscopic result of microstructural alterations that increase the susceptibility of bone to the applied load^34^ (Fig. 1).

## Osteoimmunology in hematoma and inflammatory phases

Secondary fracture healing occurs after a fracture without rigid fixation. Under the influence of active loading, an external callus is initiated to bridge the fracture gap^35^ in a three-stage process consisting of inflammation, repair, and remodeling.^36^ The first two of these partially overlapping phases restore bone structure and continuity over a period of 3 months to allow full weight bearing. The last phase involves gradual remodeling of bone to withstand the usual strains of daily life.^37^ In contrast, primary fracture healing without formation of a periosteal callus usually requires direct contact of compact bone or rigid surgical intervention that makes the fracture gap <200 μm. However, elderly osteoporotic bones, such as metaphyseal sites, which are highly susceptible to bone degradation, make it difficult to maintain anatomical reduction and rigid fixation using traditional screws due to inadequate insertional torque.^38^ In this situation, the healing process will be more like indirect bony union with the response of loading and inflammation, forming a periosteal callus bridging the fracture gap. The specific osteoimmunology and mechanosensation status of patients with osteoporotic fractures affect these healing phases in different manners.^39^

Bone fracture induces immediate inflammation and bleeding around bone extremities and within the medulla, where a template is formed for callus formation, called a hematoma.^40^ Around the hematoma sites, inflammatory cells, such as macrophages/monocytes or B/T cells, are activated to release inflammatory cytokines, including tumor necrosis factor alpha (TNF-α), interleukin-1 (IL-1), and interleukin-6 (IL-6) into the systemic circulation.^41^ These cytokines are responsible for the initiation of immune and inflammatory responses,^42^ including enhancement of blood flow and vessel permeability, as well as the recruitment of immune cells for pathogen clearance.^43^ The limited inflammatory response is required to initiate the repair cascade and mobilize all the required factors involved in the early bridging of the fracture gap, especially in indirect bony unions without rigid fixation.^44^ The interactions between the skeletal system and immune function, comprising osteoimmunology, in osteoporotic fractures are altered with age.^45^ It has been reported that an age-associated decline in the absolute numbers of human B cell precursors in bone marrow^46^ leads to a significant decrease in the number of mature human B cells.^47,48^ Compared with young adults, the B cell repertoire is less diverse in elderly individuals.^49^ As to T cells, studies exhibit reductions of proliferation and helper function in CD4+ T cells that recruit neutrophils and macrophages to infected sites of elderly individuals.^50^ Consistent with this finding, the impaired neutrophil^51^/monocytes^52^-mediated phagocytosis also showed an age-dependent reduction.^53^ In contrast, the expression of Toll-like receptors (TLRs), a group of pattern recognition receptors (PRRs) that trigger pro-inflammatory responses,^54^ is increased in monocytes and dendritic cells in elderly people, accompanied by increased production of IL-1 and TNF-α.^55^ In vitro and in vivo studies have shown that persistent tumor necrosis factor (TNF) expression impairs cell-mediated immune responses and Th2 differentiation from naïve T cells.^56–58^ Moreover, constant stimulation by TNF-α elevates the threshold for T cell activation via the T-cell receptor (TCR), attenuating T cell responses to antigen^59^ and negatively affecting angiogenesis during fracture healing.^60^ Thus, the early immune responses and pathogen clearance of aged patients with osteoporotic fractures would be impaired or delayed due to the insufficient acquired immunity and dysfunction of the innate immune system.^61^ Furthermore, pathogen infections induce host inflammation and contribute to local bone loss. The most frequent pathogen identified in bone infection is *Staphylococcus.*^62^ *Staphylococcus aureus* protein A induces the production of inflammatory cytokines, such as TNF-α,^63^ IL-6, interleukin-1 alpha (IL-1α),^64^ interleukin-1 beta (IL-1β),^64^ and neutrophil-attracting chemokines in local tissues. On the one hand, short-term (24 h) upregulated cytokines, such as TNF-α are essential for local recruitment of neutrophils,^41^ macrophages, and T cells for pathogen clearance.^65,66^ However, the long-term presence of these cytokines, especially TNF-α, IL-1, and IL-6, activates CD4+ T cells, promoting RANKL expression by osteoblasts^67^ and synergizing directly with RANK to amplify osteoclastogenesis^68^ and bone resorption.^69^

**Fig. 2:**
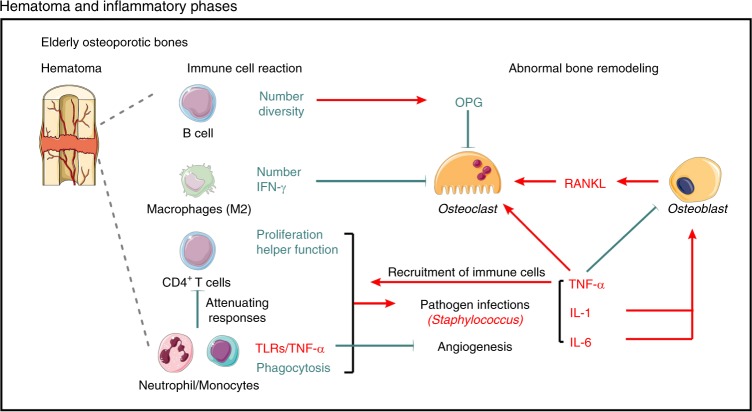
Osteoimmunology in elderly osteoporotic bones. Hematoma and inflammatory phases are the immediate reactions to a fracture. The limited inflammatory response at the fracture site is essential to initiate repair processes and mobilize all the required factors involved in the early bridging of the fracture gap, especially in indirect bony unions without rigid fixation. The high RANKL/OPG ratio caused by aging-related inflammation and the lack of mature B cells is associated with the hyperactivation of osteoclastogenesis and aggravation of bone resorption in elderly patients with bone loss, which increases the incidence of intra- or postoperative further fractures. OPG osteoprotegerin, IFN-γ interferon gamma, RANKL receptor activator of nuclear factor kappa-B ligand, TLRs toll-like receptors, TNF-α tumor necrosis factor alpha, IL-1 interleukin-1, IL-6 interleukin-6. “Red” refers to upregulation; “Green” refers to downregulation

In general, high levels of pro-inflammatory cytokines, either in the circulation or local tissues, are found in the aged population.^70^ Serum IL-1, IL-6, and/or TNF-α levels have been shown to be upregulated in elderly patients with bone loss,^70^ supporting the hypothesis of increased inflammation with aging.^71^ In fact, TNF-α promotes bone resorption by both directly inducing osteoclast differentiation^72^ and inhibiting osteoblast differentiation and function.^73,74^ IL-1 drives osteoclast differentiation via a RANKL/RANK-independent mechanism.^75^ IL-6 indirectly plays a positive role in osteoclast differentiation by binding IL-6 receptors expressed on osteoblastic cells to induce RANKL expression.^76^ Neutrophils stimulate osteoclastogenesis by upregulating cell surface RANKL expression under TLR stimulation^77^ or by inducing osteoblast retraction.^78^ Interferon gamma (IFN-γ), secreted by anti-inflammatory macrophages (M2), inhibits osteoclast differentiation via rapid degradation of TRAF6.^79^ However, macrophage polarization shows a shift toward macrophages (M1) that promote inflammatory cytokines as a consequence of aging.^80^ In contrast, mature B cells are important regulators of a decoy receptor for RANKL, osteoprotegerin (OPG). In total, 40% of the OPG in bone marrow is produced by mature B cells alone.^81^ The increased bone resorption and low levels of bone marrow OPG were demonstrated in B cell-deficient mice; this defect can be normalized by the transplantation of B cells. As a result of the decreased number of mature human B cells, the supply of OPG is low in patients with osteoporosis. Thus, current evidence supports that the high RANKL/OPG ratio caused by aging-related inflammation and the lack of mature B cells is associated with the hyperactivation of osteoclastogenesis and aggravation of bone resorption in elderly patients with bone loss, which increases the incidence of further intraoperative or postoperative fractures (Fig. 2). Moreover, Nagae et al. concluded that overactivation of osteoclasts plays an important role in chronic pain after osteoporotic fracture by creating acidosis.^82^ Hyper osteoclast activity may lead to pathological modifications of bone sensory nerve fibers, with an overexpression of acid-sensitive pain receptors, which contributes to generating and maintaining pain in osteoporosis.^83^

## Molecular basis of bone mechanosensation

Primary fracture healing occurs when the fracture site achieves rigid anatomical and mechanical fixation. Under these conditions, a soft callus enveloping the bone extremities subsequently calcifies to a peripheral solid callus by intramembranous ossification.^35^ However, in elderly osteoporotic bones, the healing process will be more like indirect bony union by forming a periosteal callus bridging the fracture gap, since it is usually difficult for the compromised bones to maintain enough stress stimulation.^84^ Among the bone multicellular units (BMUs), which consist of various cells involved in bone remodeling, the osteocytes embedded in the matrix function as major mechanosensitive cells.^85^ Substantial evidence indicates that the mechanosensation of osteocytes is mediated by signaling molecules, such as Wnts, bone morphogenetic proteins (BMPs), nitric oxide (NO), and prostaglandin E2 (PGE2) in response to mechanical stimulation.^86^ Furthermore, altered enzyme activity and RNA synthesis have been reported in osteoclasts after mechanical loading of intact bone, which further supports the mechanosensory role of osteocytes in bone.^87^ Thus, adequate mechanical loading and mechanotransduction are pivotal factors in the repair and remodeling phase of fracture healing.

Mechanical forces, including fluid flow as well as compressive/tensile forces in the LCN,^13^ induce cell-level physical signals of shear stress, electric/streaming potentials, and substrate strain by acting on cell surface sensors and within the signaling pathways.^88^ To date, evidence strongly suggests that integrins on the surface of bone cells are ubiquitous sensors of mechanical forces capable of detecting alterations in the mechanical environment in the extracellular milieu.^89^ Shear strain is detected by primary cilia via polycystin 1 (PC1) and transient receptor potential cation channel subfamily V member 4 (TRPV4), which activate signal transducer and activator of transcription (STAT) signals to induce ion flux.^90^ Wnt signaling is also activated by cilia via the noncanonical pathway, resulting in β-catenin degradation.^91,92^ The role of canonical Wnt in the suppression of the SOST gene (sclerostin) has also been demonstrated.^93^ Furthermore, fluid shear stress can activate voltage-sensitive calcium channels on the plasma membrane, leading to influx of Ca^2+^, which induces PGE2 synthesis via ATP and inhibits NO generation as a second messenger.^94,95^ PGE2 and ATP are released via connexin hemichanels formed following extracellular signal-regulated kinase1/2 (ERK1/2)-induced transcription of connexin-43 (Cx43).^96,97^ Compressive/tensile forces impose hydraulic pressure in the lacunar-canalicular system,^89^ which increases cellular deformation of osteocytes^98^. The substrate strain at the membrane can be sensed by integrins that transmit force to the cell cytoskeleton via ERK, proto-oncogene tyrosine-protein kinase Src (SRC) and replication origin activator (ROA) to induce stress fiber polymerization.^98^ The cell nucleus plays crucial roles in response to cellular mechanotransduction. Transcriptional regulation in the cell nucleus converts incoming mechanoresponsive signals into biological signaling and even directly responds to cellular deformation.^99^ These intracellular signaling pathways converge to modulate osteogenic transcription factors in addition to regulators of growth factors and matrix proteins required for osteogenesis. Evidence suggests that mechanical signals induce OPG and suppress RANKL to inhibit osteoclast differentiation.^100^

**Fig. 3:**
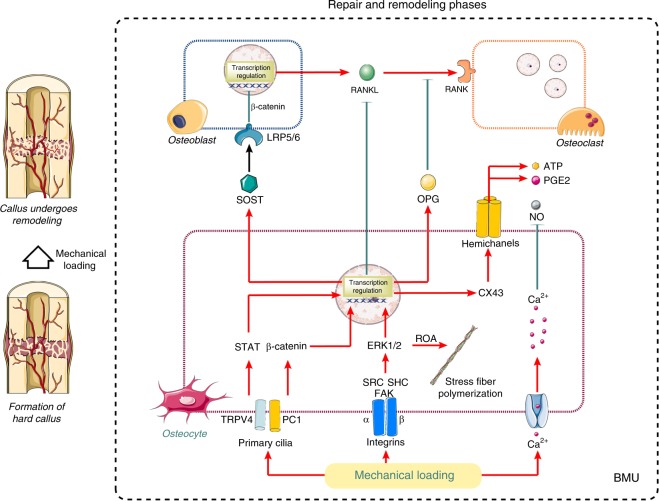
Molecular basis of bone mechanosensation. The bone multicellular unit (BMU), which consists of osteocytes, osteoblasts, and osteoclasts, functions as a large mechanosensitive organ. Mechanical loading can be sensed by primary cilia, integrins, and Ca^2+^ channels on the surface of bone cells, then transcribed in the nucleus with inhibition of RANKL production and promotion of sclerostin and OPG. LRP5/6, low-density lipoprotein receptor-related protein 5/6; SOST sclerostin, RANKL receptor activator of nuclear factor kappa-B ligand, RANK receptor activator of nuclear factor kappa-B, OPG osteoprotegerin, ATP adenosine triphosphate, PGE2 prostaglandin E2, NO nitric oxide, CX43 connexin-43, STAT signal transducer and activator of transcription, ERK1/2 extracellular signal-regulated kinase1/2, ROA replication origin activator, TRPV4 transient receptor potential cation channel subfamily V member 4, PC1 polycystin 1, SRC proto-oncogene tyrosine-protein kinase Src, SHC Shc-transforming protein, FAK focal adhesion kinase, BMU bone multicellular unit. “Red” refers to upregulation; “Green” refers to downregulation

The morphological changes of osteocytes with aging have been reported to influence their mechanosensitivity and the response to loads.^101^ Changes in LCN volume due to the increased rate of osteocytic osteolysis with aging or trauma have been shown to affect local bone mechanosensation.^102^ Additionally, age-related changes in periosteal modeling arise from cell function/signaling deficits combined with increased marrow adiposity leading to a reduced pool of osteoblast progenitors.^103,104^ Furthermore, periosteal lining cell numbers and osteoblast life-span are reduced by an increased rate of apoptosis.^105^ There is an age-related switch in macrophage differentiation from the anti-inflammatory (M2) phenotype that mediates tissue repair to the inflammatory (M1) phenotype.^53^ As a consequence of the decline in the secretion of anti-inflammatory and osteogenic cytokines, the bone regeneration capability could be impaired in the process of remodeling osteoporotic fractures.^7^ In osteoporotic fractures, the inevitable immobilization and stress shielding achieved by orthopedic surgery reduce the mechanical loading compared with that at normal sites.^106^ The deficiency of stress loading on surface sensors of bone cells is accompanied by NF-κB activation of osteoblasts and neighboring immune cells that promotes RANKL production to trigger osteoclastogenesis and bone resorption.^107,108^ This process results in the excess removal of bone mass,^93^ which therefore leads to a coarse trabecular pattern and thinning of cortical bone. Estrogen controls the adaptation of osteoblasts and osteocytes to mechanical loads via binding to the estrogen receptor (ER) or activation of TGF1 receptors.^109^ Delayed ER expression was shown to be correlated with impaired callus formation capacity in the healing process.^110^ A study in humans suggested that mechanical interventions enhance periosteal modeling and bone strength in the young skeleton,^111^ while the effects are markedly diminished in the elderly skeleton.^111,112^ In vitro studies indicate that the age-related increase in osteocyte degradation and reduction in the basal level of mechanosensation significantly affect second messenger signaling to modulate bone regeneration^111^ (Fig. 3). An optimal strategy for improving the treatment of osteoporotic fractures must address both biological and mechanical issues based on the molecular mechanisms of mechanical loading in fracture healing.^93^

## Anti-inflammatory effects of mechanical loading

**Fig. 4:**
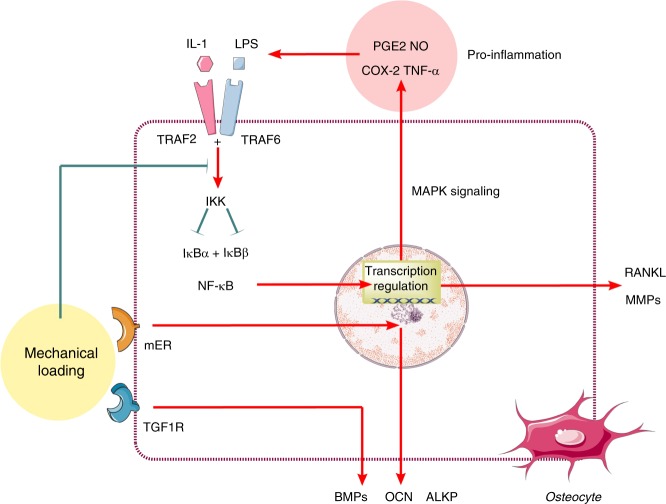
Anti-inflammatory effects of mechanical loading. The mechanotransduction at low magnitudes is a potent signal to counter inflammation activated by the NF-κB signaling cascade. IL-1 interleukin-1, LPS lipopolysaccharide, TRAF2 TNF receptor-associated factor 2, TRAF6 TNF receptor-associated factor 6, PGE2 prostaglandin E2, NO nitric oxide, COX-2 cyclooxygenase-2, TNF-α tumor necrosis factor alpha, IKK inhibitor of NF-κB kinase, IκBα NF-κB inhibitor alpha, IκBβ NF-κB inhibitor beta, BMPs bone morphogenetic proteins; OCN osteocalcin; ALKP alkaline phosphatase; RANKL receptor activator of nuclear factor kappa-B ligand, MAPK mitogen-activated protein kinase, MMPs matrix metalloproteinases. “Red” refers to upregulation; “Green” refers to downregulation

After the fracture gap has been bridged by a callus, the woven bone is slowly replaced with lamellar bone structures. Balanced resorption and formation of new bone require a normal environment without excessive inflammation.^37^ In these long-term phases, mechanics play a pivotal role, not only as the forces driving remodeling but also as the regulators that function to inhibit inflammation and abrogate the associated repression of growth factors and matrix synthesis.^113^ The traumatic signals caused by fracture and surgery initiate pro-inflammatory signaling cascades via activation of NF-κB transcription factors.^114,115^ NF-κB activation leads to the production of high levels of NO and superoxide that mediate both bone damage and matrix degradation.^116^ Mediators such as TNF-α, IL-1β, and matrix metalloproteinases (MMPs) play key roles in the pathogenesis of inflammatory bone diseases and injuries.^117^ In IL-1β-treated osteoblast-like cells, mechanical signals have been shown to rapidly (within 10 min) and dramatically inhibit NF-κB nuclear translocation.^118^ The mechanism involves the inhibition of TNF receptor-associated factor 6 (TRAF6) phosphorylation and subsequent activation of the inhibitor of NF-κB kinase (IKK) complex.^119^ This process prevents the proteosomal degradation of NF-κB inhibitor alpha (IκBα) and NF-κB inhibitor beta (IκBβ) phosphorylation, which in turn inhibits nuclear translocation of NF-κB and subsequent pro-inflammatory gene transcription.^120^ In fact, mechanotransduction at low magnitudes is a potent anti-inflammatory signal^121^ that counters the NF-κB signaling cascade.^122^ In vitro studies in osteoblasts have shown that the pro-inflammatory mediators suppressed by mechanical signals (tensile, compressive, and shear) include IL-1β-induced NO, COX-2, PGE2, cytokines (IL-1β and TNF-α), and MMPs.^123^ Simultaneously, mechanical signals upregulate the expression of growth factors, such as BMPs, OCN, and alkaline phosphatase (ALKP), which are inhibited during inflammation.^124^ Several anti-inflammatory cytokines (IL-10) and tissue inhibitors of metalloproteinases (TIMPs) that are inhibited during inflammation are upregulated by mechanical signals. For instance, IL-10 and TIMP-II synthesized by low magnitudes of mechanical signals can suppress inflammation and matrix breakdown in osteoblast and osteoblast-like cells.^125^ In contrast, exogenous PGE2 was demonstrated to function as an intercellular messenger for enhancement of the mechanosensitivity of bone to loading forces both in vitro and in vivo.^126^ Furthermore, in the presence of PGE2 signaling, osteocytes release NO in response to mechanical stimulation via cytoskeletal adaptation and mitogen-activated protein kinase (MAPK) pathways.^127^ Additionally, mechanical loading increases ER-α expression at the fracture callus, which is beneficial for mechanical signal transduction and fracture repair.^128,129^ These data indicate that rigid fixation and adequate mechanical loading are a means of improving the immune environment that benefits bone healing^130^ (Fig. 4).

## Management of hematoma and perioperative infection

**Fig. 5:**
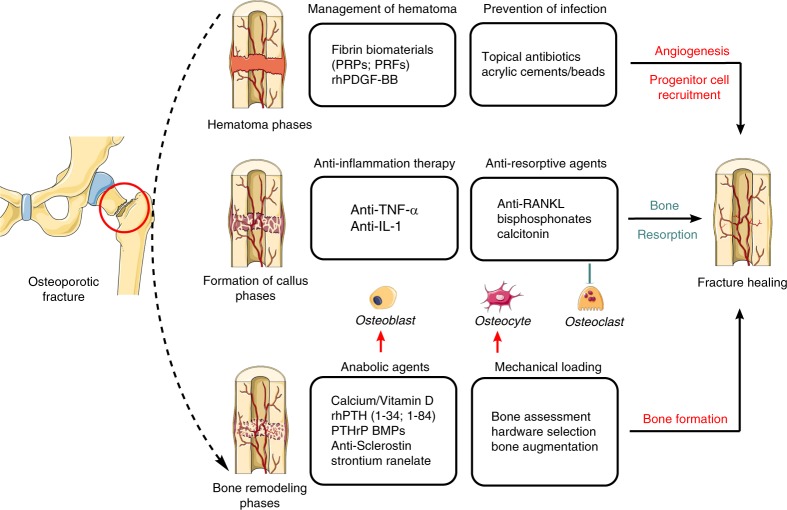
Bench-to-bedside strategies for osteoporotic fracture. The most satisfactory bone healing depends on two pivotal factors: a good biological environment and appropriate mechanical loading for bone repair and homeostasis. Bench-to-bedside strategies, including management of hematomas and perioperative infections, anti-inflammation and regulation of bone resorption, and rigid fixation and mechanical loading enhancement would benefit the creation of the proper environment for fracture healing of osteoporotic bones. PRPs platelet-rich plasma, PRFs platelet-rich fibrin, rhPDGF-BB recombinant human platelet-derived growth factor-BB, TNF-α tumor necrosis factor alpha, IL-1 interleukin-1, RANKL receptor activator of nuclear factor kappa-B ligand, rhPTH recombinant human parathyroid hormone, PTHrP parathyroid hormone-related protein, BMPs bone morphogenetic proteins. “Red” refers to upregulation; “Green” refers to downregulation

The most satisfactory bone healing depends on a good biological environment and appropriate mechanical loading for bone repair and remodeling. Orthopedic surgeons are encouraged to familiarize themselves with the molecular basis of skeletal senescence, mechanical loading and osteoimmunology in osteoporotic fractures, which is critical for determining an appropriate surgical technique or nonsurgical intervention^3^. In terms of the decrease in early immune responses and pathogen clearance in aged patients with osteoporotic fractures, special preoperative management is required to achieve a better local healing environment. Previous studies have revealed the osteoimmunological role of hematoma in fracture healing, especially in the inflammatory phase.^42,131^ However, there is still no consensus on hematoma management among surgeons. Grundnes and Reikeras reported that early removal of the hematoma (2–7 days) after fracture greatly prohibited bone healing in an animal fracture model.^132^ Other researchers, however, found that hematoma without normal fibrinolysis was an obstacle to cellular trafficking, which subsequently inhibited fracture healing by impeding macrophage accumulation.^133^ Thus, in clinical practice, early hematoma in the fracture site should be preserved and induced to “mature”. Indeed, the use of fibrin biomaterials, including platelet-rich plasma (PRPs), platelet-rich fibrin (PRFs) and other treatments,^134^ mimicking the structure of the natural hematoma, has demonstrated promising effects on the improvement of fracture healing. In addition, early-stage treatment with recombinant human platelet-derived growth factor-BB (rhPDGF-BB) could be beneficial for vascularization and angiogenesis in local sites,^135^ which would promote the recruitment of progenitors and accelerate bone remodeling in fracture healing of the elderly.^136^ In short, hematoma is a natural factor that enhances fracture healing and should be preserved in fracture sites, although thorough mechanisms are needed to be well investigated. Due to the decline of immune responses and pathogen clearance dysfunction in elderly patients with osteoporotic fractures, the prevention of perioperative infection through the use of adequate doses of antibiotics is important for maintaining a normal environment for initiating fracture healing (Fig. 5). Consequently, in orthopedic surgery, antibiotics, such as antibiotic-augmented acrylic cements/beads are increasingly used in topical form.^137^ However, the enhanced antibiotic treatment doses are thousands of times higher than those required to inhibit bacterial growth.^137^ Current evidence suggests that this concentration is detrimental to abnormal bone remodeling as a result of negative effects on mitochondrial physiology.^137^ Thus, local antibiotic vehicles must be designed to deliver sufficiently high concentrations to inhibit bacterial growth without affecting bone cell metabolism.^138^

## Anti-inflammation and regulation of bone remodeling

Increased RANKL/OPG ratios caused by aging-related inflammation are associated with hyperactivation of osteoclastogenesis and exacerbation of bone resorption in elderly patients, leading to subsequent impairment of bone healing and inflammatory pain. Anti-inflammation therapy is a potential strategy that may benefit aging-related osteoporotic fracture by reducing inflammation and providing protection against bone loss. Studies have shown that healthy transgenic mice injected with anti-TNF-α repeatedly promote T cell responses to cognate peptide antigen.^139^ In the clinical setting, anti-TNF-α (infliximab, Remicade) rapidly and remarkably restores the responses of T cells from rheumatoid arthritis (RA) patients.^56^ Treatment with infliximab protects against bone loss and improves the formation/resorption marker ratio in this population, suggesting beneficial systemic and local bone effects.^140,141^ Although anti-inflammatory therapies have not been used clinically to treat osteoporosis, they have shown good promise in mouse models. Indeed, pharmacological or genetic ablation of TNF^142^ and IL-1^143^ by somatic gene therapy^144^ has been used effectively to prevent ovariectomy-induced bone loss in mice. Thus, anti-inflammation therapy is a potential strategy that may benefit osteoporosis patients because of reduced inflammation in addition to protection against bone loss. However, the risk of infection is increased in patients undergoing anti-inflammation treatment, suggesting that anti-inflammatory drugs should be discontinued for a period of time before surgery.^145^ Because chronic inflammation affects bone healing, the anti-inflammatory drug should be reused in osteoporotic fracture after an acute immune response to alleviate inflammation-induced bone loss.

The recent development of antiresorptive agents (e.g., bisphosphonates, RANKL inhibitor) represents a significant advance in therapeutic options for improving bone quality and metabolism.^146^ Bisphosphonates are commonly used in osteoporosis to prevent and reduce pain by modifying osteoclast activity.^147^ Following an osteoporotic fracture, early intervention with anti-resorptive drugs after surgery would not affect fracture union.^148^ However, bisphosphonate-dependent repair processes become progressively dominant in the late phases, suggesting that continuous administration of alendronate causes delayed healing in mechanically compromised situations.^149^ Denosumab, a RANKL inhibitor, can significantly reduce the high RANKL/OPG ratio in the inflammatory and repair phases of fracture healing with aging-related osteoporosis^150^ and has been identified as an efficacious osteoporosis treatment option with low rates of adverse events.^151^ Calcitonin effectively relieves bone pain and can reduce bone loss in osteoporotic fractures, although short-term (3 months) use is recommended.^152,153^ In summary, reducing the frequency of postoperative syndromes in patients with osteoporosis requires not only regulation of the immune response but also balanced bone resorption and osteogenesis (Fig. 5). Studies have demonstrated that anti-inflammation therapy combined with a bone resorption blocking drug^154^ reverses systemic bone loss,^155^ while the timing and extent of immune intervention require further clinical exploration.

## Strategies for mechanical loading enhancement and rigid fixation

The biochemical responses of osteocytes to mechanical loads are mediated by signals induced via a variety of mechanosensitive proteins, such as primary cilia, integrins, and activated ion channels.^156^ However, it is as yet unclear how osteocytes perceive and differentiate responses to two drastically opposite magnitudes of mechanical signals, that is, those of physiological magnitudes that initiate regenerative responses and of traumatic signals that initiate bone damage and resorption.^87^ Appropriate use of bone formation promoters (e.g., calcium/vitamin D), mainly for osteoblasts and osteocytes, helps to further enhance the mechanical induction and repair of bone structure. Patients over 65 years old with BMD less than −2.5SD or postmenopausal women with multiple osteoporotic vertebral fractures or hip fractures who have not responded to bisphosphonate therapy should be switched to the available anabolic agents,^7,131^ including recombinant human parathyroid hormone (rhPTH,[1–34] [1–84]) and parathyroid hormone-related protein (PTHrP).^30^ Strontium ranelate is now considered effective in enhancing the biomechanical properties of bone for resistance fragility fractures. Strontium ranelate increases bone formation and decreases bone resorption, thereby rebalancing bone remodeling, which is conducive to new bone formation.^157^ Numerous studies have shown that strontium ranelates functions in improvement in all parameters related to bone quality and strength.^158^ The sclerostin monoclonal antibody, such as romosozumab, has been shown to lead to gains in hip BMD.^159^ In addition, BMPs, which belong to transforming growth factor-beta (TGF-β) family members,^160^ lead to synergistic induction of downstream TGFβ signaling for osteogenesis combined with physical microenvironment.^161^ Tricalcium phosphate and polymethylmethacrylate (PMMA) are usually employed to augment bone cement and increase the stability of implant fixation in osteoporotic bone.^162,163^ These cements undergo interdigitation in porous bone^38^ to increase the surface area of contact and provide additional resistance against the screw threads. PMMA has also been used for the delivery of drugs, such as antibiotics, via bone cements.^164^ However, PMMA undergoes an exothermic reaction during the drying process, with the potential to initiate thermal bone necrosis.^165^ In addition, PMMA is difficult to remove in cases of revision or infection without integrating into the bony architecture.^166^ In contrast, the integration of tricalcium phosphate into the bone provides a potential scaffold for biological activity and cell growth in a demineralized bone matrix.^167^ Allograft fibulas are used in bone with low BMD as tools for reduction as well as the provision of medial calcar support.^168^ As mechanical stimulation is a potent anti-inflammatory signal, sufficient postsurgery mechanical loading interventions, including physical therapy and rehabilitation, are helpful for building a supportive mechanical and biological environment around the local fracture sites for bone healing. Low intensity vibration (LIV) improves bone quality by activating cells responsible for bone remodeling and biasing the differentiation of mesenchymal and HSC progenitors toward osteoblastogenesis.^169,170^ However, current evidence is insufficient to support the benefit of ultrasound and extracorporeal shockwave therapies (ECSW) for fracture healing in clinical practice^171^ (Fig. 5).

Mechanical bone strength is vital for the stable anchorage of hardware required for fracture repair. Due to the impaired bone strength and complicated immunology environment in elderly individuals with osteoporosis, more suitable implants with better mechanical characteristics are required to improve aging-related osteoporotic fracture healing. Measurement of the thickness and porosity of cortical bone prior to surgery is important in guiding hardware selection for the repair of osteoporotic fractures. Thus, it is of great importance to identify parameters for evaluating bone quality (Fig. 5). Only 60% of the variation in bone densitometry was measured by dual-energy X-ray absorptiometry (DXA) because it is hard to recognize differences in both trabecular and cortical bone geometrical macrostructure.^172^ Both trabecular connectivity and cortical porosity significantly influence bone strength parameters, including stiffness to resist deformation and elasticity to absorb energy.^173^ To determine a better intervention, state-of-the-art clinical imaging techniques will help in measuring bone structural parameters, instead of focusing on BMD alone.^174^ Evaluation of the grayscale intensity map of DXA imaging can provide more precise information for bone structural parameters compared with BMD measurement.^175^ The trabecular bone score (TBS) correlates positively with trabecular connectivity based on evaluation of the DXA image.^176^ Combining TBS and BMD measurements provides an improved prediction of bone strength compared with BMD alone.^177,178^ Evaluations of structural, material, and mechanical properties based on bone biopsy specimens provide a reliable assessment of local bone characteristics, which are vital independent determinants of bone strength.^179^ The DensiProbe can be a helpful tool for intraoperative assessment of mechanical peak torque in mechanical testing setups,^180^ providing information that can be valuable in choosing implants. Furthermore, this approach does not increase the risk to the patient or increase the surgeon’s workload since the central peg hole can be used for the next procedure.^180^ Cortical and cancellous screws are traditional designs, with the former having relatively narrower outer diameters and decreased thread pitch.^181^ In both cases, the fixation strength depends on the torque generated between the bone and thread that resists shear.^182^ During insertion of a cancellous bone screw into the osteoporotic bone, the torque reaches the plateau prior to the contact of all the screw threads.^183^ The changes in screw geometry that confer an advantage on cancellous screws are lost below a threshold BMD of 0.4 g·cm^–1^.^184^ The plateau torque (T Plateau), which is an efficient predictor of insertion failure at the femoral head, is significantly dependent on aspects of the bone microarchitecture, such as the structure model index (SMI) and bone volume fraction (BV/TV).^185^ Previous studies suggest that a more plate-like bone structure, a higher BV/TV, and a higher surface-to-volume ratio provide a structural environment that favors cutting of the screw threads into the bone, resulting in an increased T Plateau.^186^ Unstable and comminuted fracture patterns as well as early implant-bone fatigue in osteoporotic bones lead to implant loosening and fixation failure.^3^ Locking-plate technology provides a more advantageous biomechanical environment that facilitates the formation of a fixed angle between the plate and screw.^187^ Despite the greater overall stability, locking plates may create an excessively rigid construct, which is predisposed to peri-implant fracture.^188^ In proximal humeral fractures with low BMD,^189^ computed tomography (CT) assessments suggest that locking plates do not reduce the rate of mechanical failure. In elderly patients with low BMD, tibial plateau fracture is associated with increased comminution and compromised fixation, suggesting that external fixation might be a more effective option than dual plating.^190^ An intramedullary nail (IMN) is a load-sharing device with the advantage of promoting secondary bone healing while preserving the surrounding soft tissues and minimizing fracture-induced hematomas.^191^ The loss of interlocking screw fixation can be mitigated through a number of strategies, including the application of washers and interlocking screws in multiple planes. However, cortical thinning of osteoporotic bone increases the intramedullary canal diameter, and a larger-diameter nail is required to achieve a diaphyseal fit and stability. Therefore, an early quantitative computed tomography (QCT) assessment of the cortical thickness is critical in using IMN in osteoporotic fractures. Intra-articular and complex fractures in patients with osteoporosis pose unique challenges for surgeons. These patients have inadequate subchondral bone quality to allow for anatomic reductions, and the stability of the implant is difficult to maintain after the reintroduction of weight-bearing and increased range of motion.^192^ Primary arthroplasty (total hip/knee/elbow arthroplasty) has been adopted to obtain adequate weight-bearing and early mobilization, which has a superior prognosis compared to internal fixation in acute acetabular fractures, displaced intra-articular tibial plateau fractures and complex distal humeral fractures.^193^ Despite the advent of locked anatomic plates, a majority of experts recommend arthroplasty in the context of poor bone quality and small fracture fragments (Table 1).

**Table 1:** Clinical options in osteoporotic fractures

Clinical options	Characteristic	Index	Methods	Fracture site and pattern	Disadvantage in osteoporotic bone	Ref.
Cortical bone screws	Narrow outer diameters and decreased thread pitch compared to cancellous bone screw	BMD Thickness and porosity of cortical bone	DXA QCT	Femoral heads Femoral neck fractures	50% reduction of the holding strength per 1 mm decrease of cortical thickness	^175, 194, 195^
Cancellous bone screw	Reach the plateau torque level prior to contact of all the screw threads	BMD, TBS SMI BV/TV	DXA HR-pQCT μMRI	Femoral metaphysis Distal radius Femoral heads	Reduction of thread-bone interface that produces torque	^183, 184, 196, 197^
Bicortical lag screw	Potential improvement of thread purchase	BMD, TBS SMI BV/TV	DXA HR-pQCT	Medial malleolus fractures		^185, 198, 199^
Traditional plates	Compress the fracture fragments between bone implant interface to create fixation strength	BMD Bone stiffness and strength	DXA	Regular fractures	Decrease of the axial and torsional stiffness	^190, 200, 201^
Locking plate	Fixed-angle construct between screw and plate	BMD, TBS Proximal cortical thickness Failure load	DXA QCT	Proximal humerus fractures	Reduction of callus formation without micromotion across the fracture site; Loss of fixation and screw cut-out	^5, 187, 188, 198, 202^
Intramedullary nail	Preserving the soft tissues around fracture site	BMD Cortical thickness	DXA QCT	Proximal humerus fractures	A larger-diameter nail is required to achieve a diaphyseal fit and stability	^191, 203^
Bone augmentation	Increase surface area; PMMA carries osteogenic and antibiotic drugs; Tricalcium phosphate and Allograft fibulas act more as a scaffold	BMD SMI BV/TV BMSi	DXA QCT	Femoral neck fractures Spine fractures Comminuted proximal humerus fractures	Damage surrounding soft tissues or initiate thermal bone necrosis; Difficult to remove	^38, 162– 164, 196, 204, 205^
External fixation	Lower fixation failure rates	BMD, TBS	DXA HR-pQCT	Comminution of tibial plateau fractures		^190^
Primary arthroplasty	Early mobilization and weight bearing	BMD, TBS Subchondral bone quality	DXA HR-pQCT DensiProbe	Acute acetabular fractures; Displaced intra-articular fractures of the tibial plateau		^193^

*BMD* bone mineral density, *DXA* dual-energy X-ray absorptiometry, *QCT* quantitative computed tomography, *HR-pQCT* high-resolution peripheral QCT, *μMRI* micromagnetic resonance imaging, *TBS* trabecular bone score, *BMSi* bone material strength index, *PMMA* polymethylmethacrylate, *SMI* structure model index, *BV/TV* bone volume fraction

## Conclusion and perspective

Low bone mass and compromised bone structure in osteoporotic fractures are undesirable for the reduction and rigid fixation, and the decreased regeneration and mechanosensation ability of osteoporotic bone also affect the healing. The initiation of supportive management, including anti-inflammatory drugs and sufficient application of antibiotics, is key for creating the proper environment for bone repair and homeostasis in patients with osteoporotic fractures. The adverse effects of insufficient mechanical loading in bone healing are critical factors that should be considered around the orthopedic procedure. Bench-to-bedside strategies for bone augmentation and hardware selection should be made according to further elucidation of the biomechanics and molecular mechanisms involved in bone repair. Multidisciplinary collaboration will facilitate the improvement of overall osteoporotic care and the reduction of secondary fracture incidence.

## References

[1] Brown C (2017). Osteoporosis: staying strong. Nature.

[2] Sozen T, Ozisik L, Basaran NC (2017). An overview and management of osteoporosis. Eur. J. Rheumatol..

[3] Yaacobi E, Sanchez D, Maniar H, Horwitz DS (2017). Surgical treatment of osteoporotic fractures: an update on the principles of management. Injury.

[4] Feron JM, Mauprivez R (2016). Fracture repair: general aspects and influence of osteoporosis and anti-osteoporosis treatment. Injury.

[5] von Ruden C, Augat P (2016). Failure of fracture fixation in osteoporotic bone. Injury.

[6] Smith DM, Khairi MR, Johnston CC (1975). The loss of bone mineral with aging and its relationship to risk of fracture. J. Clin. Investig..

[7] Bernatz JT (2019). Osteoporosis is common and undertreated prior to total joint arthroplasty. J. Arthroplast..

[8] Singer A (2015). Burden of illness for osteoporotic fractures compared with other serious diseases among postmenopausal women in the United States. Mayo Clin. Proc..

[9] Clark D, Nakamura M, Miclau T, Marcucio R (2017). Effects of aging on fracture healing. Curr. Osteoporos. Rep..

[10] Baxter MA (2004). Study of telomere length reveals rapid aging of human marrow stromal cells following in vitro expansion. Stem Cells.

[11] Foulke BA, Kendal AR, Murray DW, Pandit H (2016). Fracture healing in the elderly: a review. Maturitas.

[12] Turner CH (2002). Biomechanics of bone: determinants of skeletal fragility and bone quality. Osteoporos. Int.:.

[13] Florencio-Silva R, Sasso GR, Sasso-Cerri E, Simoes MJ, Cerri PS (2015). Biology of bone tissue: structure, function, and factors that influence bone cells. BioMed. Res. Int..

[14] Iwaniec UT, Turner RT (2016). Influence of body weight on bone mass, architecture and turnover. J. Endocrinol..

[15] van der Linden JC, Weinans H (2007). Effects of microarchitecture on bone strength. Curr. Osteoporos. Rep..

[16] Stock SR (2015). The mineral–collagen interface in bone. Calcif. Tissue Int..

[17] Tzaphlidou M (2008). Bone architecture: collagen structure and calcium/phosphorus maps. J. Biol. Phys..

[18] Guerado E (2016). Bone mineral density aspects in the femoral neck of hip fracture patients. Injury.

[19] Qi Z, Liu W, Lu J (2016). The mechanisms underlying the beneficial effects of exercise on bone remodeling: roles of bone-derived cytokines and microRNAs. Prog. Biophys. Mol. Biol..

[20] Katsimbri P. (2017). The biology of normal bone remodelling. European Journal of Cancer Care.

[21] Boyce BF, Rosenberg E, de Papp AE, Duong LT (2012). The osteoclast, bone remodelling and treatment of metabolic bone disease. Eur. J. Clin. Investig..

[22] Li C, Williams BO, Cao X, Wan M (2014). LRP6 in mesenchymal stem cells is required for bone formation during bone growth and bone remodeling. Bone Res..

[23] Delaisse JM (2014). The reversal phase of the bone-remodeling cycle: cellular prerequisites for coupling resorption and formation. Bone. Rep..

[24] Lai X (2015). The dependences of osteocyte network on bone compartment, age, and disease. Bone Res..

[25] Hadjidakis DJ, Androulakis II (2006). Bone remodeling. Ann. New Y. Acad. Sci..

[26] Watson EC, Adams RH (2018). Biology of bone: the vasculature of the skeletal system. Cold Spring Harbor Perspect. Med..

[27] Diab DL, Watts NB (2013). Postmenopausal osteoporosis. Curr. Opin. Endocrinol. Diab. Obes..

[28] Duque G, Troen BR (2008). Understanding the mechanisms of senile osteoporosis: new facts for a major geriatric syndrome. J. Am. Geriatr. Soc..

[29] Marie PJ (2014). Bone cell senescence: mechanisms and perspectives. J. Bone Miner. Res..

[30] Black DM, Rosen CJ (2016). Clinical practice. Postmenopausal osteoporosis. New Engl. J. Med..

[31] Yamagishi S (2011). Role of advanced glycation end products (AGEs) in osteoporosis in diabetes. Curr. Drug Targets.

[32] Chen H, Zhou X, Fujita H, Onozuka M, Kubo KY (2013). Age-related changes in trabecular and cortical bone microstructure. Int. J. Endocrinol..

[33] Osterhoff G (2016). Bone mechanical properties and changes with osteoporosis. Injury.

[34] Silva MJ (2007). Biomechanics of osteoporotic fractures. Injury.

[35] Marsell R, Einhorn TA (2011). The biology of fracture healing. Injury.

[36] Einhorn TA, Gerstenfeld LC (2015). Fracture healing: mechanisms and interventions. Nat. Rev. Rheumatol..

[37] Claes L, Recknagel S, Ignatius A (2012). Fracture healing under healthy and inflammatory conditions. Nat. Rev. Rheumatol..

[38] Rothberg DL, Lee MA (2015). Internal fixation of osteoporotic fractures. Curr. Osteoporos. Rep..

[39] Lu C (2005). Cellular basis for age-related changes in fracture repair. J. Orthop. Res..

[40] Ozaki A, Tsunoda M, Kinoshita S, Saura R (2000). Role of fracture hematoma and periosteum during fracture healing in rats: interaction of fracture hematoma and the periosteum in the initial step of the healing process. J. Orthop. Sci..

[41] Chan JK (2015). Low-dose TNF augments fracture healing in normal and osteoporotic bone by up-regulating the innate immune response. EMBO Mol. Med..

[42] Timlin M (2005). Fracture hematoma is a potent proinflammatory mediator of neutrophil function. J. Trauma.

[43] Gibon E, Lu L, Goodman SB (2016). Aging, inflammation, stem cells, and bone healing. Stem Cell Res. Ther..

[44] Briot K, Geusens P, Em Bultink I, Lems WF, Roux C (2017). Inflammatory diseases and bone fragility. Osteoporos. Int..

[45] Weng N-p (2006). Aging of the immune system: how much can the adaptive immune system adapt?. Immunity.

[46] McKenna RW, Washington LT, Aquino DB, Picker LJ, Kroft SH (2001). Immunophenotypic analysis of hematogones (B-lymphocyte precursors) in 662 consecutive bone marrow specimens by 4-color flow cytometry. Blood.

[47] Frasca D (2008). Aging down-regulates the transcription factor E2A, activation-induced cytidine deaminase, and Ig class switch in human B cells. J. Immunol..

[48] Chong Y (2005). CD27+ (memory) B cell decrease and apoptosis-resistant CD27− (naive) B cell increase in aged humans: implications for age-related peripheral B cell developmental disturbances. Int. Immunol..

[49] Weksler ME, Goodhardt M, Szabo P (2002). The effect of age on B cell development and humoral immunity. Springe. Semin. Immunopathol..

[50] Swain S, Clise-Dwyer K, Haynes L (2005). Homeostasis and the age-associated defect of CD4 T cells. Semin. Immunol..

[51] Kovtun A (2016). The crucial role of neutrophil granulocytes in bone fracture healing. Eur. Cells Mater..

[52] Hearps AC (2012). Aging is associated with chronic innate immune activation and dysregulation of monocyte phenotype and function. Aging Cell.

[53] Sinder BP, Pettit AR, McCauley LK (2015). Macrophages: their emerging roles in bone. J. Bone Miner. Res..

[54] Shaw AC, Goldstein DR, Montgomery RR (2013). Age-dependent dysregulation of innate immunity. Nat. Rev. Immunol..

[55] Qian F (2012). Age-associated elevation in TLR5 leads to increased inflammatory responses in the elderly. Aging Cell.

[56] Cope AP (1994). Chronic exposure to tumor necrosis factor (TNF) in vitro impairs the activation of T cells through the T cell receptor/CD3 complex; reversal in vivo by anti-TNF antibodies in patients with rheumatoid arthritis. J. Clin. Investig..

[57] Frasca D (2012). A molecular mechanism for TNF-α-mediated down-regulation of B cell responses. J. Immunol. (Baltim., MD: 1950).

[58] Davis LS, Cush JJ, Schulze-Koops H, Lipsky PE (2001). Rheumatoid synovial CD4+ T cells exhibit a reduced capacity to differentiate into IL-4-producing T-helper-2 effector cells. Arthritis Res..

[59] Isomaki P (2001). Prolonged exposure of T cells to TNF down-regulates TCR zeta and expression of the TCR/CD3 complex at the cell surface. J. Immunol. (Baltim., MD: 1950).

[60] Lim JC (2017). TNFalpha contributes to diabetes impaired angiogenesis in fracture healing. Bone.

[61] Oishi Y, Manabe I (2016). Macrophages in age-related chronic inflammatory diseases. Npj Aging Mech. Dis..

[62] Blanchette KA, Prabhakara R, Shirtliff ME, Wenke JC (2017). Inhibition of fracture healing in the presence of contamination by Staphylococcus aureus: effects of growth state and immune response. J. Orthop. Res..

[63] Kumar A, Tassopoulos AM, Li Q, Yu FS (2007). Staphylococcus aureus protein A induced inflammatory response in human corneal epithelial cells. Biochem. Biophys. Res. Commun..

[64] Olaru F, Jensen LE (2010). Staphylococcus aureus stimulates neutrophil targeting chemokine expression in keratinocytes through an autocrine IL-1alpha signaling loop. J. Invest. Dermatol..

[65] Stenzel W (2005). An essential role for tumor necrosis factor in the formation of experimental murine *Staphylococcus aureus*-induced brain abscess and clearance. J. Neuropathol. Exp. Neurol..

[66] Liu H (2017). *Staphylococcus aureus* epicutaneous exposure drives skin inflammation via IL-36-mediated T cell responses. Cell Host microbe.

[67] Hofbauer LC (1999). Interleukin-1beta and tumor necrosis factor-alpha, but not interleukin-6, stimulate osteoprotegerin ligand gene expression in human osteoblastic cells. Bone.

[68] Cenci S (2000). Estrogen deficiency induces bone loss by enhancing T-cell production of TNF-alpha. J. Clin. Investig..

[69] Fuller K, Murphy C, Kirstein B, Fox SW, Chambers TJ (2002). TNFalpha potently activates osteoclasts, through a direct action independent of and strongly synergistic with RANKL. Endocrinology.

[70] Scheidt-Nave C (2001). Serum interleukin 6 is a major predictor of bone loss in women specific to the first decade past menopause. J. Clin. Endocrinol. Metab..

[71] Cuturi MC (1987). Independent regulation of tumor necrosis factor and lymphotoxin production by human peripheral blood lymphocytes. J. Exp. Med..

[72] Azuma Y, Kaji K, Katogi R, Takeshita S, Kudo A (2000). Tumor necrosis factor-α induces differentiation of and bone resorption by osteoclasts. J. Biol. Chem..

[73] Gilbert L (2000). Inhibition of osteoblast differentiation by tumor necrosis factor-alpha. Endocrinology.

[74] Kitaura H (2013). Immunological reaction in TNF-α-mediated osteoclast formation and bone resorption in vitro and in vivo. Clin. Dev. Immunol..

[75] Kim JH (2009). The mechanism of osteoclast differentiation induced by IL-1. J. Immunol. (Baltim., MD: 1950).

[76] Udagawa N (1995). Interleukin (IL)-6 induction of osteoclast differentiation depends on IL-6 receptors expressed on osteoblastic cells but not on osteoclast progenitors. J. Exp. Med..

[77] Chakravarti A, Raquil MA, Tessier P, Poubelle PE (2009). Surface RANKL of Toll-like receptor 4-stimulated human neutrophils activates osteoclastic bone resorption. Blood.

[78] Allaeys I (2011). Osteoblast retraction induced by adherent neutrophils promotes osteoclast bone resorption: implication for altered bone remodeling in chronic gout. Lab. Investig..

[79] Takayanagi H (2000). T-cell-mediated regulation of osteoclastogenesis by signalling cross-talk between RANKL and IFN-gamma. Nature.

[80] Schlundt C (2018). Macrophages in bone fracture healing: their essential role in endochondral ossification. Bone.

[81] Horowitz MC, Fretz JA, Lorenzo JA (2010). How B cells influence bone biology in health and disease. Bone.

[82] Yonou H (2003). Osteoprotegerin/osteoclastogenesis inhibitory factor decreases human prostate cancer burden in human adult bone implanted into nonobese diabetic/severe combined immunodeficient mice. Cancer Res..

[83] Catalano A (2017). Pain in osteoporosis: from pathophysiology to therapeutic approach. Drugs aging.

[84] Zuo Fuxing, Xiong Feng, Wang Xia, Li Xueyuan, Wang Renzhi, Ge Wei, Bao Xinjie (2017). Intrastriatal Transplantation of Human Neural Stem Cells Restores the Impaired Subventricular Zone in Parkinsonian Mice. STEM CELLS.

[85] Tatsumi S (2007). Targeted ablation of osteocytes induces osteoporosis with defective mechanotransduction. Cell Metab..

[86] Glatt V, Evans CH, Tetsworth K (2016). A concert between biology and biomechanics: the influence of the mechanical environment on bone healing. Front. Physiol..

[87] Plotkin LI, Bellido T (2016). Osteocytic signalling pathways as therapeutic targets for bone fragility. Nat. Rev. Endocrinol..

[88] Han MKL, de Rooij J (2016). Converging and unique mechanisms of mechanotransduction at adhesion sites. Trends Cell Biol..

[89] Sikavitsas VI, Temenoff JS, Mikos AG (2001). Biomaterials and bone mechanotransduction. Biomaterials.

[90] Xiao ZS, Quarles LD (2010). Role of the polycytin-primary cilia complex in bone development and mechanosensing. Ann. New Y. Acad. Sci..

[91] Tu X (2015). Osteocytes mediate the anabolic actions of canonical Wnt/beta-catenin signaling in bone. Proc. Natl Acad. Sci. USA.

[92] Nguyen AM, Jacobs CR (2013). Emerging role of primary cilia as mechanosensors in osteocytes. Bone.

[93] Yavropoulou MP, Yovos JG (2016). The molecular basis of bone mechanotransduction. J. Musculoskelet. Neuron. Interact..

[94] Ranade SS, Syeda R, Patapoutian A (2015). Mechanically activated ion channels. Neuron.

[95] Lewis KJ (2017). Osteocyte calcium signals encode strain magnitude and loading frequency in vivo. Proc. Natl Acad. Sci. USA.

[96] Xu H (2015). Connexin 43 channels are essential for normal bone structure and osteocyte viability. J. Bone Miner. Res..

[97] Plotkin LI, Speacht TL, Donahue HJ (2015). Cx43 and mechanotransduction in bone. Curr. Osteoporos. Rep..

[98] Lynch ME, Fischbach C (2014). Biomechanical forces in the skeleton and their relevance to bone metastasis: biology and engineering considerations. Adv. Drug Deliv. Rev..

[99] Fedorchak GR, Kaminski A, Lammerding J (2014). Cellular mechanosensing: getting to the nucleus of it all. Prog. Biophys. Mol. Biol..

[100] Kim CH, You L, Yellowley CE, Jacobs CR (2006). Oscillatory fluid flow-induced shear stress decreases osteoclastogenesis through RANKL and OPG signaling. Bone.

[101] Temiyasathit S, Jacobs CR (2010). Osteocyte primary cilium and its role in bone mechanotransduction. Ann. New Y. Acad. Sci..

[102] Goggin PM, Zygalakis KC, Oreffo RO, Schneider P (2016). High-resolution 3D imaging of osteocytes and computational modelling in mechanobiology: insights on bone development, ageing, health and disease. Eur. Cells Mater..

[103] Devlin MJ, Rosen CJ (2015). The bone-fat interface: basic and clinical implications of marrow adiposity. The Lancet. Diab. Endocrinol..

[104] Li J, Liu X, Zuo B, Zhang L (2016). The role of bone marrow microenvironment in governing the balance between osteoblastogenesis and adipogenesis. Aging Dis..

[105] Manolagas SC (2018). The quest for osteoporosis mechanisms and rational therapies: how far we’ve come, how much further we need to go. J. Bone Miner. Res.:.

[106] Goodman CA, Hornberger TA, Robling AG (2015). Bone and skeletal muscle: key players in mechanotransduction and potential overlapping mechanisms. Bone.

[107] Boyce BF, Xiu Y, Li J, Xing L, Yao Z (2015). NF-kappaB-mediated regulation of osteoclastogenesis. Endocrinol. Metab..

[108] Tarapore RS (2016). NF-kappaB has a direct role in inhibiting Bmp- and Wnt-induced matrix protein expression. J. Bone Miner. Res..

[109] Thompson WR, Rubin CT, Rubin J (2012). Mechanical regulation of signaling pathways in bone. Gene.

[110] Aguirre JI (2007). A novel ligand-independent function of the estrogen receptor is essential for osteocyte and osteoblast mechanotransduction. J. Biol. Chem..

[111] Srinivasan S, Gross TS, Bain SD (2012). Bone mechanotransduction may require augmentation in order to strengthen the senescent skeleton. Ageing Res. Rev..

[112] Devlin MJ (2011). Estrogen, exercise, and the skeleton. Evolut. Anthropol..

[113] Loi F (2016). Inflammation, fracture and bone repair. Bone.

[114] Lin TH (2017). NF-kappaB as a therapeutic target in inflammatory-associated bone diseases. Adv. protein Chem. Struct. Biol..

[115] Salles MB (2015). Evaluating nuclear factor NF-kappaB activation following bone trauma: a pilot study in a Wistar rats model. PLoS One.

[116] Liu, T., Zhang, L., Joo, D. & Sun, S. C. NF-kappaB signaling in inflammation. *Signal Transduct.Target. Ther.* **2**, 10.1038/sigtrans.2017.23 (2017). 10.1038/sigtrans.2017.23 PMC5661633 29158945

[117] Kapoor M, Martel-Pelletier J, Lajeunesse D, Pelletier JP, Fahmi H (2011). Role of proinflammatory cytokines in the pathophysiology of osteoarthritis. Nat. Rev. Rheumatol..

[118] Long P, Liu F, Piesco NP, Kapur R, Agarwal S (2002). Signaling by mechanical strain involves transcriptional regulation of proinflammatory genes in human periodontal ligament cells in vitro. Bone.

[119] Agarwal S (2003). A central role for the nuclear factor-kappaB pathway in anti-inflammatory and proinflammatory actions of mechanical strain. FASEB J..

[120] Novack DV (2011). Role of NF-kappaB in the skeleton. Cell Res..

[121] Yu HS, Kim JJ, Kim HW, Lewis MP, Wall I (2016). Impact of mechanical stretch on the cell behaviors of bone and surrounding tissues. J. Tissue Eng..

[122] Pires Bruno, Silva Rafael, Ferreira Gerson, Abdelhay Eliana (2018). NF-kappaB: Two Sides of the Same Coin. Genes.

[123] Wang L (2012). Involvement of p38MAPK/NF-kappaB signaling pathways in osteoblasts differentiation in response to mechanical stretch. Ann. Biomed. Eng..

[124] Wang L (2010). Involvement of BMPs/Smad signaling pathway in mechanical response in osteoblasts. Cell. Physiol. Biochem.: Int. J. Exp. Cell Physiol., Biochem. Pharmacol..

[125] Long P, Hu J, Piesco N, Buckley M, Agarwal S (2001). Low magnitude of tensile strain inhibits IL-1beta-dependent induction of pro-inflammatory cytokines and induces synthesis of IL-10 in human periodontal ligament cells in vitro. J. Dent. Res..

[126] Sauerschnig M (2018). Effect of COX-2 inhibition on tendon-to-bone healing and PGE2 concentration after anterior cruciate ligament reconstruction. Eur. J. Med. Res..

[127] Thorsen K, Kristoffersson AO, Lerner UH, Lorentzon RP (1996). In situ microdialysis in bone tissue. Stimulation of prostaglandin E2 release by weight-bearing mechanical loading. J. Clin. Investig..

[128] Cheung WH, Miclau T, Chow SK, Yang FF, Alt V (2016). Fracture healing in osteoporotic bone. Injury.

[129] Chow SK (2016). Mechanical stimulation enhanced estrogen receptor expression and callus formation in diaphyseal long bone fracture healing in ovariectomy-induced osteoporotic rats. Osteoporos. Int..

[130] Thomas M, Puleo D (2011). Infection, inflammation, and bone regeneration: a paradoxical relationship. J. Dent. Res..

[131] Peichl P, Holzer LA, Maier R, Holzer G (2011). Parathyroid hormone 1-84 accelerates fracture-healing in pubic bones of elderly osteoporotic women. J. Bone Jt. Surg. Am. Vol..

[132] Grundnes O, Reikeras O (1993). The role of hematoma and periosteal sealing for fracture healing in rats. Acta Orthop. Scand..

[133] Yuasa M (2015). Fibrinolysis is essential for fracture repair and prevention of heterotopic ossification. J. Clin. Investig..

[134] Dohan Ehrenfest DM (2014). Classification of platelet concentrates (Platelet-Rich Plasma-PRP, Platelet-Rich Fibrin-PRF) for topical and infiltrative use in orthopedic and sports medicine: current consensus, clinical implications and perspectives. Muscles Ligaments Tendons J..

[135] Xie H (2014). PDGF-BB secreted by preosteoclasts induces angiogenesis during coupling with osteogenesis. Nat. Med..

[136] Wagatsuma A (2006). Effect of aging on expression of angiogenesis-related factors in mouse skeletal muscle. Exp. Gerontol..

[137] Kallala R (2012). In vitro and in vivo effects of antibiotics on bone cell metabolism and fracture healing. Expert Opin. Drug Saf..

[138] Antoci V, Adams CS, Hickok NJ, Shapiro IM, Parvizi J (2007). Antibiotics for local delivery systems cause skeletal cell toxicity in vitro. Clin. Orthop. Relat. Res..

[139] Cope AP (1997). Chronic tumor necrosis factor alters T cell responses by attenuating T cell receptor signaling. J. Exp. Med..

[140] Chopin F (2008). Long-term effects of infliximab on bone and cartilage turnover markers in patients with rheumatoid arthritis. Ann. Rheum. Dis..

[141] Marotte H (2007). A 1-year case-control study in patients with rheumatoid arthritis indicates prevention of loss of bone mineral density in both responders and nonresponders to infliximab. Arthritis Res. Ther..

[142] Roggia C (2001). Up-regulation of TNF-producing T cells in the bone marrow: a key mechanism by which estrogen deficiency induces bone loss in vivo. Proc. Natl Acad. Sci. USA.

[143] Kimble RB (1995). Simultaneous block of interleukin-1 and tumor necrosis factor is required to completely prevent bone loss in the early postovariectomy period. Endocrinology.

[144] Gao Y (2004). Estrogen prevents bone loss through transforming growth factor β signaling in T cells. Proc. Natl Acad. Sci. USA.

[145] Scherrer CB, Mannion AF, Kyburz D, Vogt M (2013). & Kramers-de Quervain, I. A. Infection risk after orthopedic surgery in patients with inflammatory rheumatic diseases treated with immunosuppressive drugs.. Arthritis Care Res..

[146] Hlaing TT, Compston JE (2014). Biochemical markers of bone turnover - uses and limitations. Ann. Clin. Biochem..

[147] Drake MT, Clarke BL, Khosla S (2008). Bisphosphonates: mechanism of action and role in clinical practice. Mayo Clin. Proc..

[148] Dirschl DR, Rustom H (2018). Practice patterns and performance in U.S. fracture liaison programs: an analysis of > 32,000 patients from the own the bone program. J. Bone Jt. Surg. Am. Vol..

[149] Hauser M, Siegrist M, Keller I, Hofstetter W (2018). Healing of fractures in osteoporotic bones in mice treated with bisphosphonates—a transcriptome analysis. Bone.

[150] Zaheer S, LeBoff M, Lewiecki EM (2015). Denosumab for the treatment of osteoporosis. Expert Opin. Drug Metab. Toxicol..

[151] Bone HG (2017). 10 years of denosumab treatment in postmenopausal women with osteoporosis: results from the phase 3 randomised FREEDOM trial and open-label extension.. The Lancet. Diabetes Endocrinol..

[152] Silverman SL, Azria M (2002). The analgesic role of calcitonin following osteoporotic fracture. Osteoporos. Int..

[153] Liu Y (2019). Hexapeptide-conjugated calcitonin for targeted therapy of osteoporosis. J. Control. Release..

[154] Ren L, Wang W (2018). Effect of risedronate on femoral periprosthetic bone loss following total hip replacement: a systematic review and meta-analysis. Medicine.

[155] Redlich K (2004). Repair of local bone erosions and reversal of systemic bone loss upon therapy with anti-tumor necrosis factor in combination with osteoprotegerin or parathyroid hormone in tumor necrosis factor-mediated arthritis. Am. J. Pathol..

[156] Thompson WR (2015). Osteocyte specific responses to soluble and mechanical stimuli in a stem cell derived culture model. Sci. Rep..

[157] Cianferotti L, D’Asta F, Brandi ML (2013). A review on strontium ranelate long-term antifracture efficacy in the treatment of postmenopausal osteoporosis. Ther. Adv. Musculoskelet. Dis..

[158] Iolascon G (2014). Bone quality and bone strength: benefits of the bone-forming approach. Clin. Cases Miner. Bone Metab..

[159] Langdahl BL (2017). Romosozumab (sclerostin monoclonal antibody) versus teriparatide in postmenopausal women with osteoporosis transitioning from oral bisphosphonate therapy: a randomised, open-label, phase 3 trial. Lancet.

[160] MacFarlane Elena Gallo, Haupt Julia, Dietz Harry C., Shore Eileen M. (2017). TGF-β Family Signaling in Connective Tissue and Skeletal Diseases. Cold Spring Harbor Perspectives in Biology.

[161] Rys JP, Monteiro DA, Alliston T (2016). Mechanobiology of TGFbeta signaling in the skeleton. Matrix Biol.: J. Int. Soc. Matrix Biol..

[162] Li M, Liu X, Liu X, Ge B (2010). Calcium phosphate cement with BMP-2-loaded gelatin microspheres enhances bone healing in osteoporosis: a pilot study. Clin. Orthop. Relat. Res..

[163] Hu MH (2011). Polymethylmethacrylate augmentation of the pedicle screw: the cement distribution in the vertebral body. Eur. Spine J..

[164] Bettencourt A, Almeida AJ (2012). Poly(methyl methacrylate) particulate carriers in drug delivery. J. Microencapsul..

[165] Webb JC, Spencer RF (2007). The role of polymethylmethacrylate bone cement in modern orthopaedic surgery. J. bone Jt. Surg. Br. Vol..

[166] Arora M, Chan EK, Gupta S, Diwan AD (2013). Polymethylmethacrylate bone cements and additives: a review of the literature. World J. Orthop..

[167] Watson JT, Nicolaou DA (2015). Orthobiologics in the augmentation of osteoporotic fractures. Curr. Osteoporos. Rep..

[168] Schumaier A, Grawe B (2018). Proximal humerus fractures: evaluation and management in the elderly patient. Geriatr. Orthop. Surg. Rehabilit..

[169] Chan ME, Uzer G, Rubin CT (2013). The potential benefits and inherent risks of vibration as a non-drug therapy for the prevention and treatment of osteoporosis. Curr. Osteoporos. Rep..

[170] Nagaraja MP, Jo H (2014). The role of mechanical stimulation in recovery of bone loss-high versus low magnitude and frequency of force. Life.

[171] Griffin, X. L., Parsons, N., Costa, M. L. & Metcalfe, D. Ultrasound and shockwave therapy for acute fractures in adults. *Cochrane Database Syst. Rev.* Cd008579, 10.1002/14651858.CD008579.pub3 (2014). 10.1002/14651858.CD008579.pub3 PMC7173732 24956457

[172] Miller PD (2017). The history of bone densitometry. Bone.

[173] Seeman E (2002). Pathogenesis of bone fragility in women and men. Lancet.

[174] de Bakker CMJ, Tseng WJ, Li Y, Zhao H, Liu XS (2017). Clinical evaluation of bone strength and fracture risk. Curr. Osteoporos. Rep..

[175] Silva BC (2013). Trabecular bone score (TBS)—a novel method to evaluate bone microarchitectural texture in patients with primary hyperparathyroidism. J. Clin. Endocrinol. Metab..

[176] Harvey NC (2015). Trabecular bone score (TBS) as a new complementary approach for osteoporosis evaluation in clinical practice. Bone.

[177] Shevroja E (2017). Use of trabecular bone score (TBS) as a complementary approach to dual-energy X-ray absorptiometry (DXA) for fracture risk assessment in clinical practice. J. Clin. Densitom..

[178] Iki M (2015). Trabecular bone score may improve FRAX(R) prediction accuracy for major osteoporotic fractures in elderly Japanese men: the Fujiwara-kyo Osteoporosis Risk in Men (FORMEN) Cohort Study. Osteoporos. Int..

[179] Brandi ML (2009). Microarchitecture, the key to bone quality. Rheumatology.

[180] Eckert JA, Jaeger S, Klotz MC, Schwarze M, Bitsch RG (2018). Can intraoperative measurement of bone quality help in decision making for cementless unicompartmental knee arthroplasty?. Knee.

[181] Seebeck J (2004). Effect of cortical thickness and cancellous bone density on the holding strength of internal fixator screws. J. Orthop. Res..

[182] Shea TM (2014). Designs and techniques that improve the pullout strength of pedicle screws in osteoporotic vertebrae: current status. BioMed. Res. Int..

[183] Wang T, Boone C, Behn AW, Ledesma JB, Bishop JA (2016). Cancellous screws are biomechanically superior to cortical screws in metaphyseal bone. Orthopedics.

[184] Cornell CN (2003). Internal fracture fixation in patients with osteoporosis. J. Am. Acad. Orthop. Surg..

[185] Ab-Lazid R, Perilli E, Ryan MK, Costi JJ, Reynolds KJ (2014). Does cancellous screw insertion torque depend on bone mineral density and/or microarchitecture?. J. Biomech..

[186] Karim L, Vashishth D (2011). Role of trabecular microarchitecture in the formation, accumulation, and morphology of microdamage in human cancellous bone. J. Orthop. Res..

[187] Greiwe RM, Archdeacon MT (2007). Locking plate technology: current concepts. J. Knee Surg..

[188] Miranda MA (2007). Locking plate technology and its role in osteoporotic fractures. Injury.

[189] Kralinger F (2014). The influence of local bone density on the outcome of one hundred and fifty proximal humeral fractures treated with a locking plate. J. Bone Jt. Surg. Am. Vol..

[190] Johanson NA, Litrenta J, Zampini JM, Kleinbart F, Goldman HM (2011). Surgical treatment options in patients with impaired bone quality. Clin. Orthop. Relat. Res..

[191] Ito K, Hungerbuhler R, Wahl D, Grass R (2001). Improved intramedullary nail interlocking in osteoporotic bone. J. Orthop. Trauma.

[192] McKee MD (2009). A multicenter, prospective, randomized, controlled trial of open reduction-internal fixation versus total elbow arthroplasty for displaced intra-articular distal humeral fractures in elderly patients. J. Shoulder Elb. Surg..

[193] Boraiah S, Ragsdale M, Achor T, Zelicof S, Asprinio DE (2009). Open reduction internal fixation and primary total hip arthroplasty of selected acetabular fractures. J. Orthop. Trauma.

[194] Goldhahn J, Suhm N, Goldhahn S, Blauth M, Hanson B (2008). Influence of osteoporosis on fracture fixation-a systematic literature review. Osteoporos. Int..

[195] Seebeck J, Goldhahn J, Morlock MM, Schneider E (2005). Mechanical behavior of screws in normal and osteoporotic bone. Osteoporos. Int..

[196] McAndrew CM (2018). Local bone quality measurements correlates with maximum screw torque at the femoral diaphysis. Clin. Biomech..

[197] Parkinson IH, Fazzalari NL (2008). Whole bone geometry and bone quality in distal forearm fracture. J. Orthop. Trauma.

[198] Cornell CN, Ayalon O (2011). Evidence for success with locking plates for fragility fractures. HSS J..

[199] Ricci WM, Tornetta P, Borrelli J (2012). Lag screw fixation of medial malleolar fractures: a biomechanical, radiographic, and clinical comparison of unicortical partially threaded lag screws and bicortical fully threaded lag screws. J. Orthop. trauma.

[200] Egol KA, Kubiak EN, Fulkerson E, Kummer FJ, Koval KJ (2004). Biomechanics of locked plates and screws. J. Orthop. trauma.

[201] Babhulkar S (2017). Unstable trochanteric fractures: Issues and avoiding pitfalls. Injury.

[202] Oheim R, Schinke T, Amling M, Pogoda P (2016). Can we induce osteoporosis in animals comparable to the human situation?. Injury.

[203] Sproul RC, Iyengar JJ, Devcic Z, Feeley BT (2011). A systematic review of locking plate fixation of proximal humerus fractures. Injury.

[204] Mellibovsky L (2015). Bone tissue properties measurement by reference point indentation in glucocorticoid-induced osteoporosis. J. Bone Miner. Res..

[205] Sanchez-Riera L (2010). Osteoporosis and fragility fractures. Best Pract. Res. Clin. Rheumatol..

